# Male mate choice scales female ornament allometry in a cichlid fish

**DOI:** 10.1186/1471-2148-10-301

**Published:** 2010-10-08

**Authors:** Sebastian A Baldauf, Theo CM Bakker, Fabian Herder, Harald Kullmann, Timo Thünken

**Affiliations:** 1 Institute for Evolutionary Biology and Ecology, University of Bonn, An der Immenburg 1, 53121 Bonn, Germany; 2 Zoologisches Forschungsmuseum Alexander Koenig, Adenauerallee 160, 53113 Bonn, Germany; 3 Zentrum für Didaktik der Biologie, University of Münster, Hindenburgplatz 34, 48143 Münster, Germany

## Abstract

**Background:**

Studies addressing the adaptive significance of female ornamentation have gained ground recently. However, the expression of female ornaments in relation to body size, known as trait allometry, still remains unexplored. Here, we investigated the allometry of a conspicuous female ornament in *Pelvicachromis taeniatus*, a biparental cichlid that shows mutual mate choice and ornamentation. Females feature an eye-catching pelvic fin greatly differing from that of males.

**Results:**

We show that allometry of the female pelvic fin is scaled more positively in comparison to other fins. The pelvic fin exhibits isometry, whereas the other fins (except the caudal fin) show negative allometry. The size of the pelvic fin might be exaggerated by male choice because males prefer female stimuli that show a larger extension of the trait. Female pelvic fin size is correlated with individual condition, suggesting that males can assess direct and indirect benefits.

**Conclusions:**

The absence of positive ornament allometry might be a result of sexual selection constricted by natural selection: fins are related to locomotion and thus may be subject to viability selection. Our study provides evidence that male mate choice might scale the expression of a female sexual ornament, and therefore has implications for the understanding of the relationship of female sexual traits with body size in species with conventional sex-roles.

## Background

Male traits that have evolved under sexual selection, e.g. ornaments to attract potential mates, and their exaggeration have been intensely investigated in many taxa [1,2]. However, in many species females show conspicuous ornaments as well [3]. Although female ornamentation has long been considered as non-adaptive, solely being a result of a genetic correlation to male ornaments [4], theoretical work and empirical studies suggest that trait expression in females could be promoted by male mate choice in species in which paternal investment and variance in female quality are high [5-7]. Studies addressing the adaptive significance of sexual selection in females have gained ground recently [3,8-10]. Nevertheless, evidence that male choice scales the exaggeration of a female trait is still lacking in many taxa.

Individual shape, such as morphology of ornaments or other sexual traits, is defined by the dimensions of the respective body part in relation to body size, with the scale relating trait size to body size known as allometry [11,12]. Allometric relationships are usually calculated by log-transformed allometric linear equations, Y = aX^b^, b representing the allometric slope and X and Y representing the indices of body size and trait size, respectively. Allometric relationships are classified in (a) isometry (b = 1), where the ratio of trait:body size remains constant, (b) negative allometry (b < 1), where larger individuals have relatively smaller traits, and (c) positive allometry (b > 1), where larger individuals show relatively larger traits. Allometry is suspected to evolve by three main forces: (a) evolutionary constraints, (b) natural selection, and (c) sexual selection [13,14]. Traditionally, traits under directional sexual selection are expected to show positive allometry [15]. Striking examples for positive allometry are extraordinarily exaggerated structures, such as antlers of the Irish elk, *Megaloceros giganteos *[16] or the horns of different beetles species [e.g. [15,17,18]]. In contrast to the traditional view, Bonduriansky [12,14] suggested that both sexual and natural selection may produce a range of allometric relationships depending on net selection on body size and traits. For example, dedicated secondary sexual traits may indeed show positive allometry, but sexually selected modifications to structures with important viability-related functions may exhibit isometric or even negative allometric scaling due to conflicting sexual and viability selection. Consequently, the absence of positive allometry does not necessarily imply that a trait is not affected by sexual selection.

Allometric relationships of male traits and their homologous expressions in females have been investigated in a wide range of species [e.g. [14] and citations therein, [19]]. However, this aspect is less well examined in species in which females show distinct sexual traits that are not expressed by males, for example many (biparental) fish species. Whereas empirical studies now repeatedly have shown that female ornamentation is subject to male sexual selection even in species with conventional sex-roles [e.g. [20-22]] knowledge about the impact of male choice on female trait allometry and the extent to which female ornamentation signals benefits to males and is still scarce. Lebas et al. [23] provided one of the few examples that allometry of a female ornament in the dance fly, *Rhamphomyia tarsata*, might signal fecundity to males.

In general, surprisingly few studies deal with trait allometry in fishes but see [24], although in many fishes the sexes show exaggerated ornaments like elongated, colorful fins, which are assumed to have resulted from sexual selection [1]. For example in swordtail fish, *Xiphophorus helleri*, females prefer males that show a sword-shaped fin [25]. In cichlids, the role of either male or female fin size in mate choice is largely unknown. Moreover, potential sexual ornaments in female cichlids, such as the size of the female pelvic fin, are solely considered to be important for comfort movements [e.g. [26]] or egg placement [27], and thus assumed to be not socially significant.

In the present study we examined the allometry and sexual selection of a female sexual trait in the biparental, socially monogamous cichlid fish, *Pelvicachromis taeniatus*, in which the female pelvic fin is a conspicuous sexual ornament. Both sexes of *P. taeniatus *show such ornaments, as well as other sexual traits. Females develop exceedingly large pelvic fins, which also differ from male fins in shape and color: females exhibit a triangular fan-shaped fin, whereas males develop a long thread-shaped fin. During courtship, both sexes present their nuptially colored ventral body region by arching it towards the partner, while intensely quivering the whole body. Simultaneously, females fan out their violet pelvic fin, thereby enlarging their violet ventral nuptial projection area, suggesting that the fin is actively used during mate choice. The female pelvic fin might function as an exaggeration of the female's ventral nuptial coloration area, which acts as a quality signal in intersexual communication (SAB, TCMB, HK, TT unpublished data).

Recent studies have shown that both sexes of *P. taeniatus *are choosy during mate choice: close kin are preferred over non-kin [28,29] and larger mating partners over smaller ones [30]. Furthermore, females of *P. taeniatus *prefer yellow nuptially colored males over dull males [31], and males prefer females that show a larger extent of violet nuptial coloration. Thus, *P. taeniatus *seems to be a well-suited model system concerning male choice and its impact on female trait allometry.

**Figure 1:**
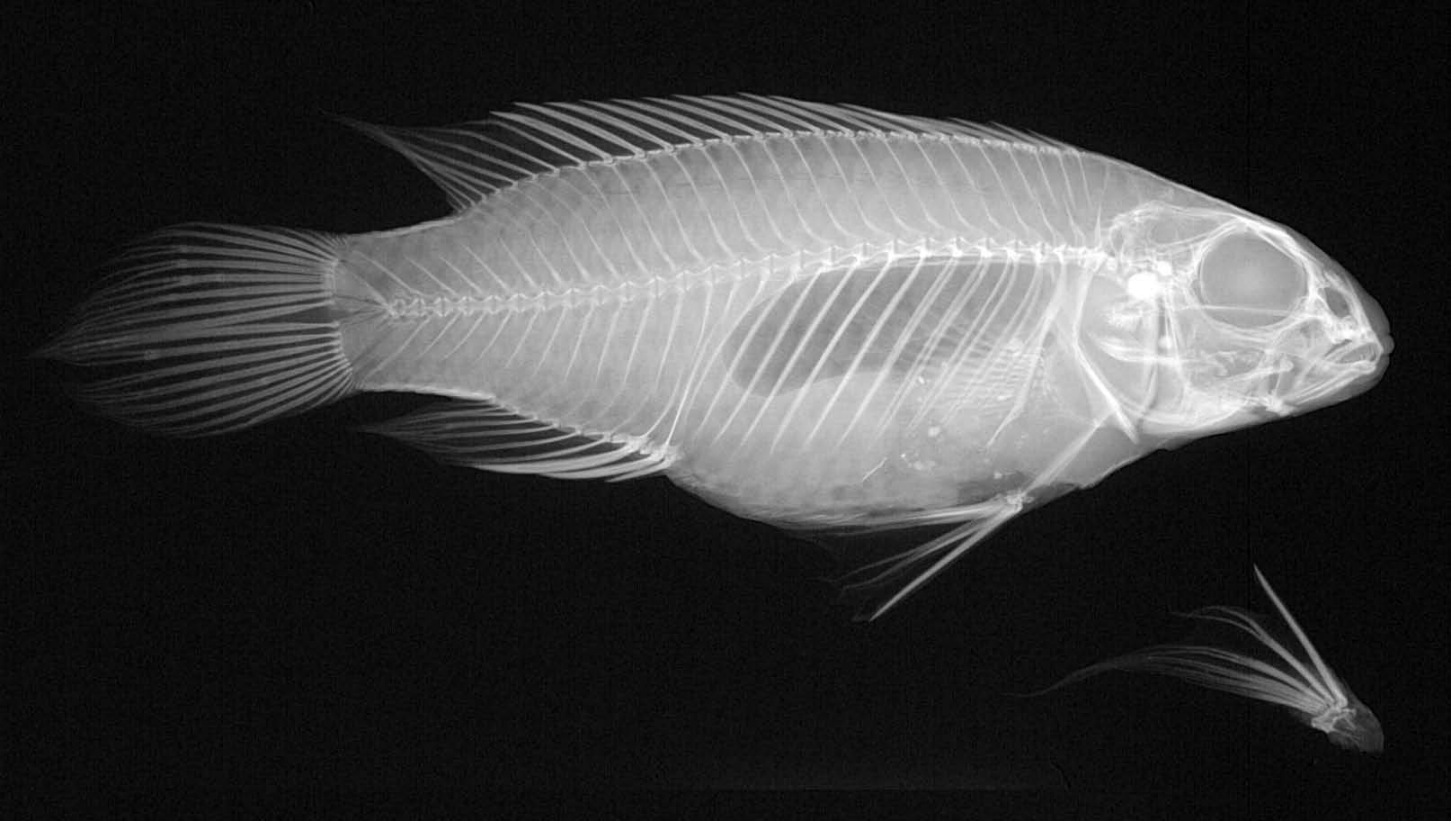
**Sample of an X-ray image**. Example of an X-Ray image of a female *P. taeniatus*. One of the paired pelvic fins was dissected in order to avoid errors in measurements by overlap in X-Rays.

First, we investigated the allometric relationship of the pelvic in comparison to the other fins, i.e. anal, caudal, dorsal, and pectoral fin. Estimates of body and fin sizes were based on measurements of bony structures. For this purpose, X-ray images of females (see Figure 1) that greatly differed in body size were analyzed. Second, we experimentally tested male preferences for females that showed larger or smaller pelvic fin size in order to test whether it is currently influencing male mate choice. To achieve this we conducted a series of mate choice experiments using computer animations of females that artificially differed in the size of the pelvic fin. A striking advantage of computer-manipulated stimuli is a high degree of standardization between the stimuli, thus minimizing the effects of confounding variables like rapid changes in coloration or different responses in stimuli fish [31-33]. Although computer systems and their visual displays are tailored to human vision they have been shown to be an appropriate method to investigate preferences in our study species concerning the perception of movement, shape and coloration [31].

## Results

### Fin allometry

**Figure 2:**
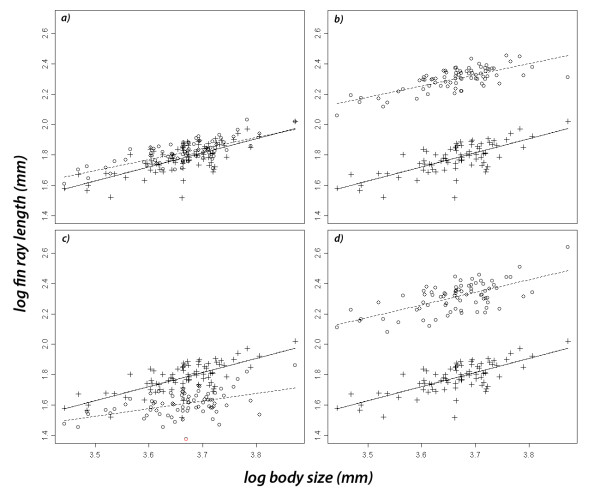
**Differences between allometric slopes of fins**. Pairwise comparisons of the allometric slopes between the pelvic fin (crosses, solid lines) and other fins (dots, dotted lines): a) anal fin, b) pectoral fin, c) dorsal fin, d) caudal fin.

The size of each fin group was highly significantly related to body size (Pearson and Spearman rank correlations: all r > 0.48, all p < 0.001). However, allometry significantly differed between fin groups (full model lrt: interaction between fin groups and body size, χ^2 ^= 17.97, p = 0.0012). Post-hoc tests revealed that the pelvic fin differed significantly in allometry from the anal fin (lrt: χ^2 ^= 6.24, p = 0.012; Figure 2a), and highly significantly from the dorsal fin (lrt: χ^2 ^= 12.69, p < 0.001; Figure 2c). Pelvic fin's allometry tended to be different from that of the pectoral fin (lrt: χ^2 ^= 3.76, p = 0.052; Figure 2b), however, was not significantly different from that of the caudal fin (lrt: χ^2 ^= 0.72, p = 0.39; Figure 2d). The size of the pelvic fin was significantly related to individual body condition (Spearman rank correlation: n = 79, r = 0.31, p = 0.004).

The pelvic and the caudal fin were both isometric because their allometric slopes did not significantly differ from 1 (Table 1). In contrast, the relationship between fin size and body size was negatively allometric for of the anal, dorsal and pectoral fin (Table 1) because their slopes were significantly smaller than 1.

**Table 1:** Allometric slope of fins

Trait	slope	S.E.	t	p
pelvic fin	0.9297	0.1022	-0.69	0.49
caudal fin	0.829	0.1123	-1.52	0.13
anal fin	0.736	0.07816	-3.38	0.001**
pectoral fin	0.7361	0.0764	-4.62	< 0.001***
dorsal fin	0.5012	0.108	-3.45	< 0.001***

Allometric slope with standard error (S.E.) of 79 female fish and statistical test (test statistic t and probability p) whether the allometric relationship between fin size and standard length deviated from 1. *** p < 0.001, ** p < 0.01.

### Male mate choice experiment

**Figure 3:**
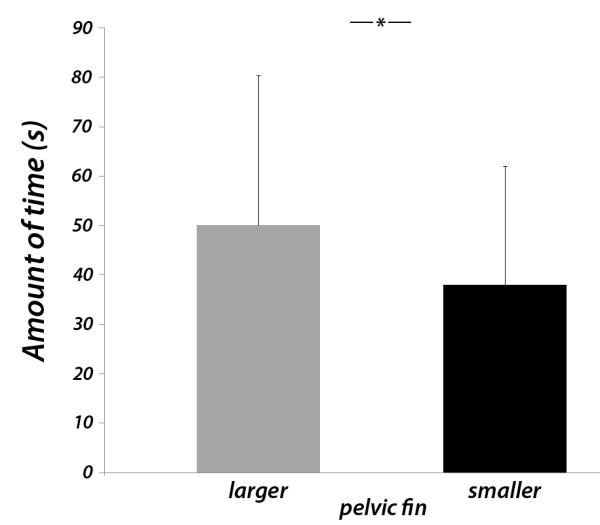
**Males preferences**. Male preferences for female stimuli that presented different sizes of the pelvic fin (100% vs. 50%, 50% vs. 0% and 100% vs. 0% pelvic fin size). The amount of time (means + S.D.) males spent in the association zones in three experimental treatments with the female stimulus that either showed a larger or a smaller pelvic fin size is shown. A linear mixed effect model was fitted (see the text for details). * p < 0.05.

Males significantly preferred the female stimulus showing a larger pelvic fin (lrt: n = 18, df = 1, χ^2 ^= 5.33, p = 0.02; Figure 3). Neither body size of males (lrt: n = 18, df = 1, χ^2 ^= 1.18, p = 0.28) nor differences in stimulus size (lrt: n = 18, df = 1, χ^2 ^= 0.15, p = 0.93) had any significant effects.

## Discussion and Conclusion

Our study is one of the first to investigate the allometry of a conspicuous female ornament, in this case the size of the female pelvic fin in a cichlid fish. The results demonstrate that the allometry of the female sexual ornament (pelvic fin) is scaled more positively than that of the other fins. Male preference for a larger female pelvic fin size in *P. taeniatus *suggests that sexual selection in females might have influenced female trait allometry.

### Fin allometry

The pelvic and caudal fins show isometry in relation to body size, whereas the anal, dorsal and pectoral fins show negative allometry: females exhibit a constant ratio of pelvic or caudal fin size to body size, whereas the other fins are relatively smaller in larger females. Furthermore, significant differences between the slopes of log-log regressions of the female fins support differences in their allometric relationships. The strongest differences in slopes occur between the pelvic fin in comparison to the anal, dorsal, and to a lesser extent to the pectoral fin. The caudal fin showed a similar relative growth in relation to body size. Thus, the size of the pelvic and caudal fin is more positively related to body size than the other fins. These results imply that the female pelvic, as well as the female caudal fin, underlie other selective pressures than the other fins. The result of the male choice experiment suggests that the female pelvic fin size might have been exaggerated by male choice.

Negative allometric relationships (b < 1) are usually thought to be characteristic for non-sexually selected traits [34,35], whereas sexually selected traits should exhibit positive allometry (b > 1). Thus our finding of an isometric relationship in a sexually selected trait contradicts these traditional views on the effect of sexual selection on trait allometry [e.g. [15]]. However, Bonduriansky and Day [12] specify a more complex relationship between selection on body size and traits by incorporating viability selection. Natural and sexual selection could have synergistic effects on the evolution of traits, thus sexually selected traits may be scaled into isometry or even negative allometry. Recent studies have demonstrated that sexually selected characters do not need to exhibit positive allometry [36,37]. Furthermore, House and Simmons [38] suggested that the amount of variation in traits under directional sexual selection may be limited by natural selection. Such constraints might explain the absence of positive allometry in a trait that is related to locomotion or other viability related functions. In *P. taeniatus *natural selection might constrain the pelvic fin size because oversized pelvic fins may lower the predator escape response of this fish. Thus, the isometric growth might be a result of sexual selection constrained by natural selection acting on the size of the pelvic fin.

The caudal fin allometry was not significantly different from that of the pelvic fin. The selective pressures acting on caudal fin size were not object of the present study. In other fishes a range from negative to positive allometric relationships of the caudal fin size has been reported [39-41]. Recent work in other fish species suggests that natural selection, e.g. predation, influences caudal fin morphology [42-45]. For example, in western mosquitofish, *Gambusia affinis*, fish originating from populations which are subject to high levels of predation exhibit a larger caudal fin than those from predator-free populations, resulting in greater maximum burst-swimming [43]. Nevertheless, sexual selection cannot be ruled out as a selective force that might scale the size of the caudal fin as well like in guppies, *Poecilia reticulata *[46]. Although the disentanglement of natural and sexual selection acting on different fins is difficult, future studies could address whether changes in allometry between fins might appear, for example under different environmental conditions. Artificial selection experiments or measurements of phenotypic plasticity could address whether environmental conditions, such as predation regime or water-flow speed, might have different impacts on fin allometry. On the other hand, mate choice experiments could elucidate the role of sexual selection acting on the size and allometry of different fins.

### Male mate choice experiment

Males associated more often with female stimuli showing a larger pelvic fin size. Why are male *P. taeniatus *sensitive to female pelvic fin size? Female ornaments could act as indicators of female quality [47]. Males may benefit from being choosy through (a) direct benefits, for example deriving from female fertility, fecundity or the amount of maternal care, or (b) from indirect, genetic benefits for their offspring [2], deriving from enhanced viability or parasite resistance or enhanced attractiveness of daughters [48,49]. Our results show that pelvic fin size of female *P. taeniatus *is positively related to body condition, thus suggesting that the size of the female pelvic fin reveals individual quality: females with high body condition may show a decreased mortality risk through disease or starvation during brood care [50], and sire daughters that are more attractive to males of the following generation. Thus, male *P. taeniatus *may get both direct as well as indirect benefits when choosing females that show pronounced expression of the quality signal.

The pelvic fins are colored similar to the ventral violet nuptial belly coloration of a female. The extent of the violet ventral coloration is associated with female quality revealing female fecundity, readiness to spawn, maternal quality and offspring-survival (SAB, TCMB, HK, TT unpublished data). Consequently, the pelvic fin could honestly enhance the transfer of quality information about an individual, and males could expect direct fitness benefits when choosing a female that shows a larger pelvic fin. However, the trait might be used to exploit male preferences, especially when the expression of the trait may imply low costs. Nevertheless, the positive correlation between pelvic fin size and individual condition contradicts the exploitation hypothesis, but future studies need to address whether the pelvic fin as well as its coloration honestly signals female quality and the heritability of the trait.

In addition to male choice, intrasexual female-female competition may be important for the evolution of females' pelvic fin size. In *P. taeniatus*, like in many other cichlids [26], females show sequential aggressive behavioral patterns towards other conspecific females. Before a fight escalates females form their body into an S-shape to threaten a potential rival. Here, the female pelvic fin is fanned out to enlarge the lateral projection area. Hence, a larger pelvic fin size might imply advantages during female-female competition, for example when the decision whether to attack or escape from a rival is estimated by a larger lateral display area. Thus, the size of the pelvic may evolve by both inter- and intrasexual selection.

Female showiness expressed by morphological traits, such as conspicuous ornaments, is widespread in many taxa. Moreover, recent research suggests that male choice occurs far more often than expected [51]. Our study is, to our knowledge, the first to show that male choice might scale the allometry of a female sexual trait, and therefore has implications for the understanding of the scaling relationship of female traits with body size.

## Methods

### Fin allometry

#### Experimental animals

Animals used for measurements were adult lab-bred fish raised under standardized laboratory conditions. The parents of the fish originated from the Moliwe river in Cameroon (04°04'N/09°16'E), West Africa. The fish were a mixed stock from different cohorts, thus varying substantially in body size. Prior to measurements, the fish were kept in eight different aquaria (50 × 30 × 30 cm). The water was tempered at 25°C and a 12:12 h (light:dark) cycle was provided to resemble natural conditions. Fish were fed daily with a mixture of frozen *Chironomus *larvae and *Artemia *spp. The measurements were conducted between April 20^th^, 2009 and May 25^th^, 2009. Altogether, 79 females were measured for the allometric examinations. The study conforms to the legal requirements of Germany for the use of animals in research.

#### Fin measurements

Fish were caught in random order from the tanks. In order to avoid damage of the fins, each female was carefully caught with a hand net, and its body mass was measured immediately. However, damages of fin tissue resulting from intra-specific conflicts can affect the estimate of fin size based on fin area. Therefore, we measured fin size as the length of fin rays. Fin ray length was highly significantly related to fin area (see Table 2). Fin area was calculated by analyzing a standardized digital photograph of each fish (including a size scale (mm^2^) in the scene) that was taken immediately before pelvic fin dissection. The fins were fanned out orthogonal to the body and fixed with a needle. The fin area (in mm^2^) was then estimated by counting the number of pixels of each fin and setting the value in relation to the number of pixels that made up the size standard (Sigma Scan pro 5).

**Table 2:** Relationship between fin ray length and fin area

trait	mean ± SD (mm)	t or S	r	p
pelvic fin	5.95 ± 0.57	32485	0.6	< 0.001***
anal fin	6.16 ± 0.47	7.77	0.66	< 0.001***
caudal fin	10.13 ± 1.00	39564	0.52	< 0.001***
dorsal fin	5.00 ± 0.41	45505	0.45	< 0.001***
pectoral fin	10.0 ± 0.75	8.48	0.69	< 0.001***
body size	39.07 ± 2.91	-	-	-

Mean ± S.D. of fin ray length for various fins and body size (n = 79), the test statistic (t or S), the correlation coefficient (r) and its significance (p) for relationship between fin ray length and fin area. *** p < 0.001.

In order to avoid damage to the fins fish were shock-frozen by placing them into saltwater that was cooled down to a temperature of -14°C. After taking the photograph the pelvic fin was dissected. The fish body and the dissected fin were preserved in ethanol (70%). The fish were X-rayed together with their dissected pelvic fin in a digital X-ray device (Faxitron Digital Specimen Radiography System LX-60) with an integrated digital camera (Figure 1). A size scale was installed during all X-rays.

Fin ray length was taken as proxy for fin size, measured to the nearest micrometer from digital X-ray-images using the software ImageJ (version 1.41; [52]). Pelvic fin length was measured as length of the pelvic spine from the X-rays of the dissected pelvic fin. In case of the anal fin, length of the 3^rd ^(the longest) anal spine was quantified. Likewise, length of the 17^th ^(last and longest) dorsal spine was measured. Caudal fin length was estimated by calculating a mean value from the four middle caudal fin rays (the longest caudal rays in *P. taeniatus*). Total body size was measured as standard length from the upper jaw symphysis to the end of the hypural plate, based on the X-ray-images. Pectoral fin ray length was measured with a digital calliper directly from the preserved fish, measuring the longest fin ray. Individual body condition was calculated as a function of body mass and standard length ((100 × mass)/standard length^3 ^[53]).

#### Statistical analysis

Parametric Pearson correlation tests were applied when data were normally distributed according to Shapiro Wilk tests, otherwise non-parametric Spearman rank correlation tests were used. All metric variables in models were log transformed and were graphically inspected for normality by using normal quantile plots of the log regressions. For analysis we used R 2.9.1 software package [54]. Linear mixed effect models ("lme", package "nlme" [55]) were fitted to measure differences in allometry between fin groups. Fin size was used as dependent variable, fin type as fixed factor and female body size as covariate. Female identity was entered as a random factor to account for the fact that the fins were not independent from each other. A significant interaction between fin groups and body size would reveal that the fins differ in their allometry. Thus, we first fitted a full model including all fin groups and tested the interaction with body size. Second, post-hoc pairwise comparisons between the pelvic fin and the other fin types concerning their allometric relationships were conducted by testing whether the slopes were significantly different from each other. Reported p-values refer to the increase in deviance in model fit when the respective variable was removed (likelihood-ratio-tests ("lrt")).

To test whether the relationship between the size of each fin and body size were isometric, negatively or positively allometric, we used a procedure analogous to Student's t-test, testing whether the allometric slopes were significantly different from one. Given p-values are two-tailed throughout. P-values < 0.05 were considered statistically significant.

### Male mate choice experiment

#### Experimental animals

All individuals of *P. taeniatus *were bred and maintained under standardized laboratory conditions. The parents of the test subjects originated from the Moliwe river in Cameroon (04°04'N/09°16'E), West Africa. All 18 test individuals were derived from the F1 generation sired from 18 unrelated pairs and were raised in mixed-sex family tanks (80 × 30 × 30 cm). The tanks were surrounded with opaque plastic sheets to avoid visual contact to other aquaria. Test fish were 1-2 years old and reproductively active. The water temperature was kept at 25 ± 1°C and natural light conditions were given (light/dark 12/12 h). Nutrition was provided once a day with a mixture of frozen *Chironomus *spp. and *Artemia *spp.

#### Preparation of artificial stimuli and experimental design

Artificial stimuli like computer animations provide a high degree of experimental standardization. However, computer displays are tailored to human vision and do not emit wavelengths such as ultraviolet or polarized light [32,56], which may play a role in the vision of shallow cichlid species [57]. Hence, it might be possible that artificial computer animations are perceived differently from the way real fishes would appear in the natural environment. However, previous studies have shown that *P. taeniatus *reliably perceives computer stimuli concerning movement, body shape and coloration displayed by a cathode-ray-tube monitor [30,31].

A digital photograph (Olympus Camedia Widezoom 5060) of a nuptially colored female was taken to obtain source data for a two-dimensional fish model. Images were saved in RAW-format to avoid the loss of coloration data due to algorithmic compression. They were white-balanced during import to Adobe Photoshop CS3. To achieve animations of the models we used "The GIMP 2.20 with animation package". A grey background image (1024 × 400 px) was created (RGB: 238, 238, 238) including a plant as a reference object in the middle of the background. Each animation consisted of 30 frames per second, which is an established method to present artificial stimuli to our test species [32]. Each stimulus moved a horizontal pathway from one side of the monitor to the other for a period of 15 seconds, including a two second stop in the middle. After that, the stimulus recurred horizontally and moved back in the same time frame. We created three different experimental treatments showing female stimuli differing in pelvic fin size (100% vs. 50%, 50% vs. 0% and 100% vs. 0% pelvic fin size). Each treatment was conducted twice with stimuli shown on switched monitor sides. Thus, each male received six experimental trials, which were conducted in random order.

Experiments were conducted between January 12^th^, 2009 and February 20^th^, 2009. Males were randomly chosen and individually isolated in separate tanks (30 × 20 × 20 cm). The mating readiness of each test fish was determined visually on the basis of the ventral coloration as well as the display of courtship behavior in the family tanks [28,29]. The isolation tanks were surrounded by print-outs of the animation's background at the broad sides and Styrofoam at the longer sides, thus ensuring that fish did not interact with other isolated individuals and were able to habituate to the background and the reference object. In each habitat aquarium a breeding cave was installed. All other conditions were similar to those of the mixed-sex tanks. Test fish were transported to the experimental set-up in their isolation tank, thus reducing stress by leaving the fish in its familiar habitat.

The set-up was illuminated by a fluorescent tube (37 W) installed one meter above the middle of the tank. Additionally, white Styrofoam surrounded the set-up. The habitat aquarium containing the test fish was placed between two CRT monitors of the same model (EIZO Flex Scan F520, 85 Hz, connected to a Matrox G550 PCIe graphic board). An association zone of 5 cm in front of each monitor was marked on the white Styrofoam under the tank creating a 20 cm neutral zone in between.

During an acclimatization period of 15 minutes both screens showed the background. After acclimatization, the stimuli appeared simultaneously on both monitors [58]. Fish behavior was recorded using a webcam. After all experimental treatments the standard length of the test fish was measured. A naïve observer analyzed the video recordings. Mating preferences were measured as association time near a stimulus of the opposite sex, which reliably predicts mating decisions in *P. taeniatus *[28]. The time spent in each association zone was calculated over a period of two minutes after the fish had first visited an association zone. For each test fish, we averaged the time spent in front of each stimulus in the first and the second trial of each treatment, thus excluding possible side biases.

#### Statistical analysis

Data were tested for normality with Shapiro Wilk tests and analyzed by fitting a linear mixed effect model ("lme", package: "nlme" [55]), with the time in front of the stimuli as dependent and stimulus type (larger or smaller pelvic fin) as explanatory variable, and male identity as random factor. Furthermore, body size and treatment (100% vs. 50%, 50% vs. 0% and 100% vs. 0% pelvic fin size) were included into the model to reveal whether these factors have an impact on male choice. Significant differences between treatments would suggest that larger differences between stimuli translate into stronger male preference.

## Authors' contributions

SAB participated in the design of the study, collected data, performed statistical analyses and wrote the paper. TCMB, FH and HK participated in the design of the study and the writing of the paper. TT participated in the design of the study, statistical analysis and the writing of the paper. All authors read and approved the final manuscript.
